# If My Memory Serves Me Well: Investigating My Memory for the Past 24 Years

**DOI:** 10.5334/joc.334

**Published:** 2024-01-19

**Authors:** Gert Storms

**Affiliations:** 1 Faculty of Psychology and Educational Sciences, University of Leuven, Tiensestraat 102, B3000 Leuven, Belgium

**Keywords:** Autobiographical memory, Long-term memory, Memory

## Abstract

This paper reports on a study of my autobiographical memory for 2691 notes recorded over 24 years in my diary, without any intention to ever use the notes as test material. I never read any of the notes again until the start of the memory study. I remembered less than two thirds of the recorded events and the retention curve showed a curvilinear shape. I dated 2% of the described events correctly but misdated on average about one and a half year, with an equal number of over and underestimations of the event age. Retention correlated significantly with ratings of salience, emotional involvement, pleasantness, event rehearsal and self-relatedness, but not with intimacy. Dating accuracy correlated with salience, pleasantness, intimacy and event rehearsal, but not with emotional involvement or self-relatedness. Regression analyses showed that event rehearsal was the best predictor of retention and dating, but the predictive value of other ratings was dependent on the content of the recorded events.

## Introduction

Autobiographical memory is a fundamental cognitive process that constitutes the basis of who we are. As William James phrased it: if someone awakes one morning with all personal memories erased, he or she would essentially be a different person (James, 1890, as cited in Wilson & Ross, 2003). Autobiographical memory is of crucial importance in forming and adjusting the self-concept (Conway, 2005; Erikson, 1950; Prebble, Addis, & Tippett, 2013; Wilson & Ross, 2003). It also has an important social function, as communicating about personal memories facilitates the development, maintenance and strengthening of social bonds (Neisser, 1988; Nelson, 2003). Furthermore, autobiographical memory helps in solving problems and developing opinions and attitudes, since recalling events from the past often offers relevant information for the present and may even predict future behavior (Pratt, Arnold, Norris, & Filyer, 1999). Accurately recalling personal memories is also vital in many practical situations, for instance, in witness testimonies (Loftus, 1979), or when producing anamnestic information during medical or psychological treatment (Moss & Goldstein, 1979).

Because of its importance, both the accuracy (and failure) and the different functions of autobiographical memory have been studied extensively in experimental and developmental psychology (e. g., Bluck, 2003; Conway & Pleydell-Pearce, 2000; Fivush, 2011; King, Romero, Schacter, & St. Jacques, 2022; Matsumoto, Watson, Fujino, Ito, & Kobayashi, 2022). Research has also focused on individual differences in autobiographical memory (Rubin, 2021) and its role in clinical populations (Barry, Hallford, & Takano, 2021; Raes, Hermans, de Decker, Eelen, & Williams, 2003).

It is generally acknowledged that long-term memory research that focused on recall for facts and universally experienced events, like famous persons, political decisions or television shows, provides limited insight into memories for events in an individual person’s life (Catal & Fitzgerald, 2004). Another common practice, often employed in experiments designed to specifically study autobiographical memory, is eliciting personal memories by randomly chosen or specifically selected cue words. This methodology was introduced by Galton (1879) when he studied his own memory. However, when participants in laboratory studies execute such tasks, events with a strong emotional content may be suppressed (Wagenaar, 1986), thereby affecting the ecological validity of the results. Furthermore, one cannot objectively control when, or even whether, the described events did in fact really occur. (See Loftus, 2004, for convincing evidence showing the malleability of memory.)

Another procedure, one that guarantees the veracity and correct dating of the investigated autobiographical events, is the diary method. In diary studies, subjects write down events that occur during a particular time. These notes are then used to study the accuracy and completeness of memories for these events at a later time. However, if the participants are college students, as in Betz and Skowronski (1997) and Walker, Vogl and Thompson (1997), for instance, the memory period under study is usually relatively short. Some researchers therefore chose to study their own memory. The advantage of this procedure is that much larger retention intervals can be investigated and the researcher can be assured of the motivation of his (single) participant. However, the number of researchers who studied their own memory in this way is surprisingly small. A crucial question then, is to what extent the results can be generalized to other people. All researchers who conducted such a single participant diary study have addressed this issue and I will come back to this criticism in the discussion section. (See also Sotgiu, 2021.)

The single participant diary method was pioneered by Madorah Smith (1952), who used her personal and her mother’s diaries to question her memory from the age of 3 onwards until the age of 63, when she conducted the study. More than two decades later, Marigold Linton (1975, 1978) recorded daily, during a six year period, two or more personal events, 5500 records in total, which she used as test material at a later time, focusing on retention of the date of the event. The chronologically next study was White (1982, 1989, 2002, 2020), who recorded one personal event daily during a year and tested his memory after 1, 2, 6, 20, and 40 years. In another diary study, Wagenaar (1986) daily recorded a personal event by the keywords ‘what’, ‘when’, ‘where’ and ‘who’. He questioned his memory by systematically varying these four types of information as cues. Larsen (1992) and Berntsen (1999; in Sotgiu, 2021) also described diary experiments to investigate their autobiographical memories, but limited their studies to a period shorter than 1 year. Finally, Catal and Fitzgerald (2004) describe a study based on notes of daily events (not discussing personal experiences), kept by the first author for over 20 years. She questioned her own and her husband’s memory (i.e., the second author) for these events when they both were in their late seventies.

The above described single participant diary studies, varied in a number of aspects: retention time, the number of recorded events, the criteria used to select the specific events, the statistical procedures used to analyze the data, the dependent variable that was the focus of the study, etc. Importantly, with the exception of Smith (1952), the diary notes were kept with the intention of studying the memory of the investigator. As a result of these differences, the findings from the studies are sometimes unequivocal. The shape of the retention curve, for instance, was linear in Linton (1978), but curvilinear in Catal and Fitzgerald (2004), Wagenaar (1986) and White (2002).

The effect of a series of variables was investigated in some, but not in all diary studies. Wagenaar (1986), for instance, reported significant relations of remembering with salience, pleasantness, emotional involvement and event rehearsal. White (2002) found a significant effect for the former three, but not for event rehearsal, while that variable was shown to affect memory in Catal and Fitzgerald (2004) and Linton (1978). Also, White (2002) failed to find a significant effect of self-relatedness. The resulting blurred picture that arises warrants clarification, preferably in an analysis that reveals *independent* contributions of these variables.

Finally, Wagenaar’s (1986) and Catal and Fitzgerald’s (2004) diary studies have shown that *what* exactly happened in a remembered event forms the core of the corresponding memory. However, the content of memories can diverse hugely. ‘A passionate kiss’, to take an example of a studied event from Linton (1978), differs on a wide range of variables from a professional dispute or a reflection on a public event, like the attacks on the twin towers in New York. To my knowledge, none of the diary studies focused on possible differences in content dependent memories.

In this paper, I describe a study in which I investigated my memory for personal events of a period of 24 years, as described in my diary. Unlike most single participant diary studies, I wrote my diary without any intent to ever use it as a test of my memory. Also unlike most comparable memory studies, the notes contained a considerable number of very personal details. I never read back any of the notes that I made of this period until the start of the study. My main goals were to find out (1) to what extent I still remember these personal events, (2) how well I remember the time when these events occurred, (3) which characteristics of the events predict the retention quality and the dating, and (4) whether autobiographical memories can be considered a homogeneous collection of memories, or whether my memory works differently depending on the remembered content (like memories of travels, family members, books read, work-related events etc.).

## Method

In this section, I will first describe how the events were recorded and how they were presented to me to test my memory. Next, I will describe the recall and scoring process. I will end this Method section with some background information on the only participant in the study (i.e., me).

### Recording of the Events

When I was 22 years old (September 1982), I started keeping a diary, a habit that I still have today. This involves noting events, as well as reflections and considerations, some of them of a very personal nature, on a regular basis. I write ‘whenever I feel like it’, that is, oftentimes many days in row and even several times a day, but also on many days I don’t take any notes. There have even been a few times when I did not make any notes during months in a row. Until December 1997, I wrote the diary notes on paper, in notebooks. All notes were dated and, from the beginning, I took up the habit of separating notes of different events or reflections on different subjects, written down on the same day, by a blank line. On the 8^th^ of January 1998, when I was 37, I started writing down my notes on my personal computer and I kept the blank line separation habit. Texts that were separated by a blank line or written down on different dates will be called ‘entries’ in the rest of this paper.

### Presentation of the Entries

When the idea of using my diary notes to question my memory occurred to me, I looked for a way to present the different entries in a random order, without date information and without the help of another person.^1^ Therefore, a program was written^2^ that takes a Word document as input, cuts it up in the different entries, and presents entries one by one in a randomized order.

**Figure 1:**
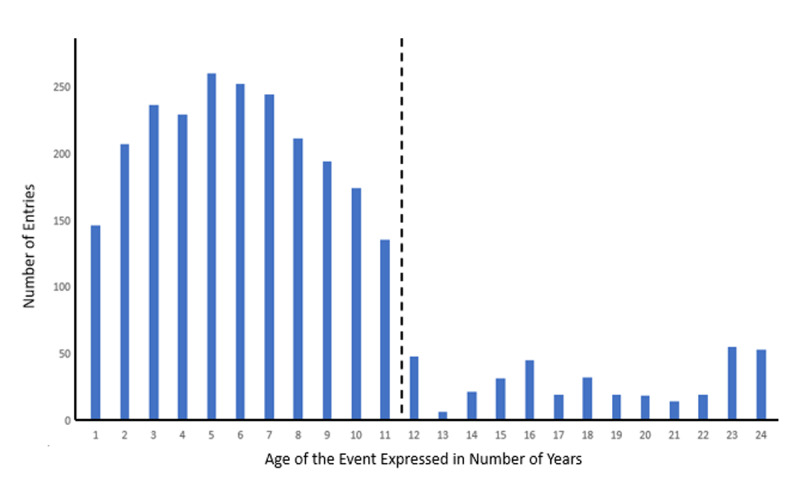
Number of Entries as a Function of the Age of the Events Expressed in Number of Years.

The advantage of using my diary is, as mentioned above, that all entries were written down without any intention to study my autobiographical memory. The disadvantage, of course, is that the material grew unsystematically and had to be taken ‘as it is’. The nature of the entries cannot be more specifically described than as ‘whatever I deemed important enough to write down at that moment’. In total, 2691 entries were used (i.e., one every 3.25 days, but see below for the frequency distribution over the covered period). Some were written down elaborately, while others were very concise and written in a hurry. The average length was 217.25 words. The shortest and the longest was, respectively, 18 and 2730 words long. Importantly, my diary activity was not equally divided over the 24 years, as Figure 1 clearly shows. Between 1998 and 2009, I wrote down notably less entries than after 2010 (and before 1998), probably because in the first years covered by the study, we moved to a country house that needed a considerable renovation and my wife and I, both professionals, raised little children (born in 1998, 2000 and 2002) during this time.

**Figure 2:**
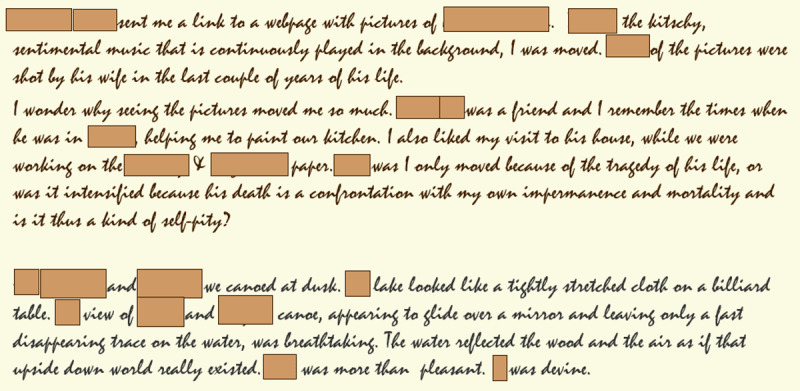
Two examples of entries as presented during the memory study.

It was not possible to control my recollection systematically for critical details, as Wagenaar (1986) did.^3^ However, for a number of the entries, I could verify a ‘critical detail’ if it contained proper person or place names beginning with a capital letter. To make this possible, the application presented the text of an entry with all words beginning with a capital letter masked. The application could of course not distinguish between proper names and other first words of a sentence, both of which began with a capital letter in the text. However, to be able to read the sentence, as well as to check whether I remembered the mentioned persons or places, the masks could be removed by clicking on the word. For two examples of entries as they were presented, see Figure 2.

### Recall and Scoring Process

I started the memory testing on the 30^th^ of January 2021 and completed all entries on the 7^th^ of February 2022. Similar to Wagenaar’s (1986) and Linton’s (1978) experiences, recalling and dating what was described in the entries appeared to be unexpectedly exhausting and it was not always easy to motivate myself to search for memories of experiences that varied in importance (or sometimes in triviality). On days when I worked on the task (almost always in the evening), I tried to finish at least 10 entries and succeeded at most to finish 20. On average, I completed 7.25 entries per day.^4^

Apart from trying to remember what was described in the entry and dating it, I rated the events for a number of variables that are prominent in theories of autobiographical memory, a procedure comparable to other single participant diary studies. For every presented entry, I wrote down the following in a spreadsheet:

1. The (randomized) entry number, which was used to identify the exact date of the entry when analyzing the data at the end of the study.
2. Date of recall (needed to calculate the exact age of the memory at recall).
3. All-or-none remembrance: whether I remembered what was written down in the entry, scored dichotomously (1 or 0).
4. Gradual Retention Score: the extent to which I remembered what was written down in the entry, rated on a 7-point scale. This was motivated because in a try out on entries written down in my diary before 1998 (i.e., entries that are not included in the study and that were written down in notebooks), it was often not easy to decide whether I did or did not remember the event, since it appeared not an all or none matter. The 7-point rating scale has a midpoint of 4, meaning that higher ratings were rather well-remembered, while values below 4 were used for relatively vague memories. Similar ratings were used by Catal and Fitzgerald (2004) and White (1982).
5. Whether I correctly inferred a place name that was masked upon first reading (1 or 0).^5^
6. Whether I correctly inferred a person name that was masked upon first reading (1 or 0).
7. Estimated date of the entry event: my best estimate of the precise date when the entry was written.
8. My judgement of how certain I was about the estimated date, rated on a 3-point rating scale, with 1 and 3 meaning low and high confidence, respectively. A similar procedure was used by Linton (1975).
9. Whether the entry could be considered an event (possibly with a reflection or consideration added to it) or whether it was merely a reflection or consideration.
10. To which of 16 categories the entry belonged (most) content wise. The categories were chosen after re-reading parts of the notes from my diary notebooks (i.e., entries not used in the memory study) with the goal to divide the test material over different content domains that, in hindsight, seemed important to me while keeping the diary during decades. The list of the 16 categories and the corresponding number of events (and percentage) classified is displayed in Table 1. It turned out that every entry could be categorized in one of them.
11. Rated salience of the event on a 7-point scale (1 = very common event that could happen daily; 2 = weekly; 3 = monthly; 4 = yearly; 5 = once every 5 years; 6 = once every 15 years; 7 = once in a life time). The way I scored the events was not so much based on actual frequency, but rather on the importance of the event. Common events therefore usually, but not necessarily, resulted in lower scores and uncommon events that left a larger impression lead to higher scores. Salience (or importance) was rated in a similar way in several single participant diary studies before (Catal & Fitzgerald, 2004; Linton, 1975; Wagenaar, 1986).
12. Rated emotional involvement on a 7-point rating scale (1 = very low and 7 = very high emotional involvement). A similar scale was used in single participant diary studies by Linton (1975) and Wagenaar (1986).
13. Rated pleasantness on a 7-point rating scale (1 = very unpleasant, 7 = very pleasant). Pleasantness ratings were also gathered in single participant diary studies by Wagenaar (1986) and White (1982) and is deemed important by several experts on autobiographical memory (e.g., Conway, Collins, Gathercole, & Anderson, 1996).
14. Rated intimacy on a 7-point rating scale (1 = not intimate at all, 7 = very intimate). This variable has been shown to be important in autobiographical memory (Conway & Playdell-Pearce, 2000; McAdams, 1982).
15. Rated event-rehearsal on a 7-point rating scale (1 = the event was very seldom talked about or ruminated of, 7 = the event was very frequently talked about or ruminated of). Event-rehearsal was rated in a similar way in single participant diary studies by Catal and Fitzgerald (2004), Linton (1975) and Wagenaar (1986).
16. Rated self-relatedness on a 7-point rating scale (1 = not related to myself at all; e.g., public events and related reflections, 7 = strongly related to myself). Ratings of Self-relatedness were also used by White (1982) and it is deemed important in Conway’s theory on autobiographical memory (e.g., Conway, 2005; Conway, Collins, Gathercole, & Anderson, 1996; Conway & Pleydell-Pearce, 2000).

**Table 1:** Content Categories of the Entries and Their Share in the Complete Data Base.


CONTENT CATEGORY	NUMBER OF ENTRIES (%)

Work	541 (21.3 %)

Literature	329 (12.9 %)

Health/Existential Meaning	247 (9.7 %)

Traveling	261 (10.3 %)

My children	260 (10.2 %)

Public Events	141 (5.5 %)

Friends from Leuven	137 (5.4 %)

My Wife	125 (4.9 %)

Dreams	118 (4.6 %)

House/Neighbors	104 (4.1 %)

Extended Family	94 (3.7 %)

Movie/TV/Theater	83 (3.3 %)

Music	58 (2.3 %)

Weather/Nature	54 (2.1 %)

Friends from my Hometown	48 (1.9 %)

Ex Romantic Partners	46 (1.8 %)


Note that, unlike in Wagenaar’s (1986) study, for instance, the values of the rating scales were given when trying to remember the described events, not at the time when the entries were written down (which was usually not long after the events occurred). This is a crucial difference, because some events may have been very important, relevant or emotional when they happened, but may have left a much more superfluous impression years later (e. g., a quarrel with a friend or a nightmare).

Whether the memory task was a recognition or a retrieval task essentially depends on how much information about the events was presented. With few exceptions, the entries contain no detailed descriptions of exactly what happened. Concise delineations are given, usually accompanied by reflections and considerations, as is illustrated by the two examples presented in Figure 2. The all-or-none judgments and the gradual retention scores reflect whether I could really dig up a memory trace of the described event that went beyond the limited information read in the diary entry. Therefore, I am inclined to label the task as retrieval rather recognition, though the boundary between those two processes, easily distinguishable in experimental research, is not so clear in the context of my study, or any of the other single participant diary studies.

### Participant

My age was 60 when I started the memory experiment and 61 when I completed the data gathering. During that time, I was employed as a full professor in cognitive psychology at the faculty of psychology and educational sciences of the University of Leuven, Belgium. My research was mainly on semantic memory, combined with a special interest in research integrity. I am married and I have three adult children. No changes occurred in these conditions during the time of the memory experiment.

## Results

### Dependent Variables

All of the analyses described below are based on entries that referred to events. Thus, all entries that merely describe a consideration or reflection that was in no way related to a specific event were excluded from these analyses. I did, however, also analyze the complete data set, which included these mere reflections or consideration, but the results of both analyses were virtually identical.

The three main dependent variables were the all-or-none retention variable, the 7-point gradual retention variable, and the absolute time estimation error (expressed in number of days), which was calculated as the absolute value of the difference between the estimated date of the entry event and the time the event occurred. I remembered 1625 of the 2542 entries, which amounts to 63.9 %. The average score on the 7-point gradual retention variable was 3.31 (with a standard deviation of 1.86). For the entries that were considered forgotten when scored dichotomously, the average gradual retention score was 1.22 (standard deviation 0.52). The entries that were not forgotten when scored dichotomously yielded an average of 4.51 (standard deviation 1.13) on the 7-point gradual retention rating scale.

The average absolute time estimation error equaled 545 days (with a standard deviation of 1101). Fifty-seven entries (2.24%) were dated exactly correct. The age of the entries was overestimated 1289 times (50.7%) and underestimated 1196 times (47.1%). On average the age of the entries was slightly overestimated with 26.9 days. Over all entries, there was a significant, but modest correlation of 0.17 between the age of the entry and the dating error.

### Reliability and Validity of the Ratings

To estimate the reliability of the six 7-point rating scales, a random sample of 50 events was rated again in April 2023, that is, more than a year after finishing the memory testing. The test-retest correlation of salience, emotional involvement, pleasantness, intimacy, event-rehearsal, and self-relatedness was, respectively, 0.48, 0.69, 0.73, 0.62, 0.72, and 0.39. Thus, especially the ratings of salience and self-relatedness showed rather low reliability.

Although I did not distinguish between entries that had no masked (i.e., capitalized) person and place names on the one hand, and entries for which I made an error in trying to infer the target name (see Footnote 5) on the other hand, I hypothesized that my memory should have been better for entries where I correctly filled in a capitalized word. This hypothesis was generally confirmed. Entries for which I correctly inferred a person as well as a place name (N = 502) resulted in an average rating of 5.26 on the 7-point gradual retention scale and an average absolute dating error of 297.8 days. Entries for which I only correctly inferred a person name and entries for which I only correctly inferred a place name (N = 58 and N = 85, respectively) yielded average gradual retention scores of 5.81 and 4.94, respectively, and average absolute dating errors of 255.7 and 354.5, respectively. The corresponding values for the remaining 1897 entries were 2.66 and 627.4.

As a final check, I hypothesized that I would better remember the entries that were related to an event than entries that were merely reflections or consideration (i.e., in no way related to a specific event). This hypothesis was also confirmed. The average score on the gradual retention scale for the ‘mere reflections/considerations’ was 1.55 (N = 149), while the average of the event entries (with or without reflections/considerations; N = 2543) was 3.32. The corresponding absolute time estimation errors were 848.2 and 544.7 days.

### Meta-cognition on Estimated Dates

I correlated the certainty ratings about the estimated date of the events (rated on a 3-point rating scale) with the absolute dating error. The correlation equaled –0.44 (p < .001), showing that my time estimation was indeed more accurate as I was more confident about the estimate.

### Retention Curve

**Figure 3:**
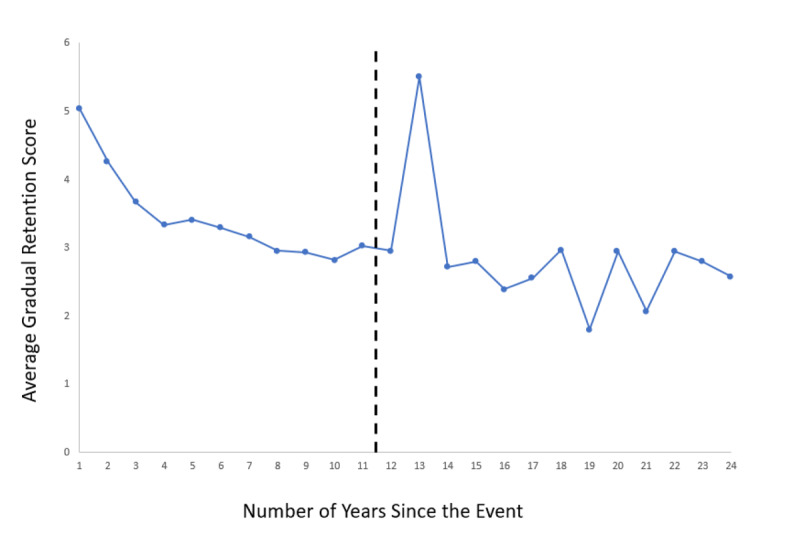
Average Retention Rating on the 7-Point Scale as a Function of the Years Passed Since the Event.

Figure 3 shows the retention curve, expressing the average value on the gradual retention scores for all 24 years covered by the study. An ANOVA on all 2542 data points with the age of the event expressed in number of years past as independent variable, showed a significant linear trend (F(1, 2518) = 161,8, p < .001). However, the quadratic trend was also significant (F(1, 2518) = 77.9, p < .001), indicating rapidly, non-linear forgetting. Because the data base contained relatively few entries for the period older than 11 years and because there is a clear outlier for the events of 13 years before the memory task (which is based on only 4 entries), I repeated the analyses on the last 11 years before the start of the memory test only (see the dashed line in Figures 1 and 3). Again, both the linear and the quadratic trend yielded significance (F(1, 2164) = 168.7, p < .001 and F(1, 2164) = 41.6, p < .001, respectively), corroborating the findings of the complete data set.

I performed similar analyses on the absolute dating error. An ANOVA on all 2542 data points with the age of the event expressed as number of years past (over all 24 years) as independent variable showed a significant linear trend (F(1, 2518) = 75.3, p < .001), but again the quadratic trend was also significant (F(1, 2518) = 30.7, p < .001). The analyses based only on the last 11 years before the start of test showed that the linear and the quadratic trend were again both significant (F(1, 2164) = 66.1, p < .001 and F(1, 2164) = 8.0, p < .005, respectively).

### Correlations

Table 2 shows the correlations between the three dependent variables, the entry length, the age of the entry at the time of recall, and the six rating scales. The correlations between the all-or-none retention variable with all other measures is almost always lower than the corresponding correlations with the 7-point gradual retention scores. This finding can be seen as evidence for the gradual nature of forgetting: remembering an event from the past is not an all or none matter. Because of the corresponding higher correlations, the remaining retention analyses will be based on the gradual retention scale, rather than on the all-or-none retention variable.

**Table 2:** Correlations Between Dependent Variables, Entry Length, Entry Age, and the Rating Scales.


	0/1	1/7	EST.ERR.	LENGTH	AGE	SAL.	EMOT.	PLEAS.	INTIM.	EV.REH.

1/7	0.86*									

Est.err.	–0.25*	–0.28*								

Length	0.20*	0.24*	–0.11*							

Mem. Age	–0.17*	–0.24*	0.17*	0.02						

Salience	0.12*	0.17*	–0.10*	0.16*	0.19*					

Emot. Involv.	0.08*	0.16*	**–0.04**	0.23*	0.01	0.22*				

Pleasantness	0.19*	0.23*	–0.07*	**–0.05**	0.12*	**–0.04**	**–0.04**			

Intimacy	**–0.05**	–0.02	**0.04**	**0.06**	0.09*	0.11*	0.49*	0.03		

Event rehear.	0.42*	0.51*	–0.20*	0.25*	**0.04**	0.29*	0.41*	0.28*	0.07*	

Self Relatedness	**0.05**	0.09*	–0.03	0.14*	0.06*	0.19*	0.70*	0.17*	0.52*	0.33*


*Note*: 0/1 is the all-or-none retention variable. 1/7 is the gradual retention score. Est.err. refers to the absolute time estimation error. Length is the number of lines of the entry. Mem.Age is the age of the memory, expressed in days, that is, the number of days past between noting the entry and testing my memory for it. Salience, Emotional involvement, Pleasant, Intimacy, Event Rehearsal, and Self Relatedness are the rating on the 7-point Likert scales. Values in bold are significant at p < .05 level and values with an asterisk a p < .01 (2-tailed, N = 2542).

The best predictor of both the gradual retention scores and the absolute dating error is, by far, how often the event has been talked or ruminated about since writing down the entry: event rehearsal explains more than 25% of the variability in the gradual retention variable and slightly less than 4% of the variability in the absolute dating error. However, both the entry length, the age of the event, the rated pleasantness and the rated salience yield correlations significant at the .01 level for both dependent variables. Emotional involvement and self-relatedness also correlate significantly with the gradual retention variable, but do not correlate significantly with the absolute dating error. Intimacy is the only rating scale that does not correlate with the gradual retention scores, though it correlates modestly but significantly (at the .05 level) with the absolute dating error.^6^

The above described correlations of the ratings with dependent variables should be interpreted cautiously, however, because of their intercorrelations. Emotional involvement has a lot of variability in common with intimacy, event rehearsal, and self-relatedness and to a lesser extent with salience. Self-relatedness further correlates significantly with intimacy, salience, and event rehearsal and to a lesser extent, but significantly, with pleasantness. Furthermore, event rehearsal also correlates relatively high with pleasantness and with salience.

### Regression Analyses

To further study the unique contributions of the different predictor variables in explaining gradual retention and the absolute dating error, regression analyses were calculated. Predicting the gradual retention scores from the entry length, event age and the six rating variables, yielded an adjusted R^2^ value of 0.38. All predictor variables contributed significantly, except for emotional involvement and intimacy.

A similar regression analysis that predicted the absolute dating error yielded a much lower adjusted R^2^ value of 0.08. Entry Length, event age, pleasantness, event rehearsal and salience contributed significantly, while emotional involvement, intimacy and self-relatedness did not.

#### Separate Analyses of the Content Categories

I ran two times 16 similar regression analyses, for the entries of every content category separately. Table 3 shows the average gradual retention scores (and standard deviation) and the R^2^ values, as well as which of the predictor values contributed significantly, with gradual retention as dependent variable. Note, however, that some of these regressions were run on rather small sets of data points. Keeping the generally accepted rule of thumb of 10 data points per predictor variable in mind, regressions on data from the categories music (N = 58), weather/nature (N = 54), friends from my hometown (N = 48) and ex romantic partners (N = 46), should be considered less reliable.

**Table 3:** Mean Gradual Retention Scores, R^2^ and Significant Predictors of the Regression Analysis with Gradual Retention as Dependent Variable.


	TOTAL SET	WORK	LITERATURE	HEALTH/MEANING	MY CHILDREN	TRAVELLING	PUBLIC EVENT	FRIENDS LEUVEN	MY WIFE	DREAMS	HOUSE/NEIGHBORS	EXTENDED FAMILY	MOVIE/TV/THEATER	MUSIC	EX ROMANTIC PARTNER	WEATHER/NATURE	FRIENDS/HOMETOWN

Mean	3.32	3.59	3.56	2.59	2.87	4.25	3.43	3.98	2.7	1.39	3.66	4.4	3.73	3.91	3.17	1.46	4.02

St.Dev.	1.85	1.85	1.15	1.86	1.88	1.7	1.7	1.78	1.76	1.02	1.86	1.67	1.82	1.77	1.77	1.01	1.66

R squared	0.38	0.38	0.29	0.36	0.45	0.43	0.34	0.33	0.48	0.35	0.33	0.43	0.61	0.53	0.45	0.55	0.49

Adjusted	0.38	0.37	0.27	0.35	0.43	0.41	0.31	0.29	0.44	0.3	0.28	0.37	0.57	0.45	0.33	0.46	0.39

Entry Length	•	•		•	•		•	○									

Memory Age	•	•	•	•	•	•		•	•	•	•	•	•	•		•	

Rehearsal	•	•	•	•	•	•	•	•	•	•	•	•	•	•		○	•

Salience	•	•				•											

Emot.lnv.					•	○							○			○	

Pleas.	•	•				○	•					○	•		•		

Intimacy					•									○		•	

Self-relat.	•	○	•			○									○		


*Note*: Significant contributions at the .01 level and at the .05 level in the regression are indicated, respectively with the symbols • and ○.

It is obvious from the first row of Table 3 that there is a large variability in the extent to which I remember events described in the entries of the different categories. Average gradual retention values differed from 1.39 for dreams to 4.25 for entries in the travel category. Likewise, the extent to which the gradual retention values can be predicted by the 8 predictor variables varies widely, with adjusted R^2^ values ranging between 0.27 for the literature (i.e., books read) category to 0.57 for the movie/TV/theater-related entries. Note also that the average gradual retention values for the 16 categories did not correlate with the corresponding percentage of explained variance (i.e., the adjusted R^2^ values), r = –.01.

Looking at the variables that contribute to the prediction of the gradual retention scores, it is remarkable that the length of the entry, that is, the amount of information presented in the entry, yields significance in less than half of the regression analyses. More important seems the age of the memory. Unsurprisingly, the more recent an entry, the higher the gradual retention score. Only one of the six rating scales contributed almost always significantly in the prediction, namely event rehearsal. The other rating scales yielded significance in a limited number of analyses. Even pleasantness contributed significantly in only 7 out of the 16 regression analyses. Furthermore, the patterns of significant contributions seem to be unsystematic, suggesting that autobiographical memories relating to different contents are stored in different ways.

Table 4 shows the results for the regression analyses with the absolute dating error as dependent variable. The mean absolute dating errors again vary widely over the different content categories, with values ranging from 269 days for entries related to my children, to 1364 days for notes on dreams. As could be expected from the relatively low correlations of the rating scales with absolute dating error, the adjusted R^2^ values in the fourth row of Table 4 are much lower than the corresponding values in Table 3. There are a few exceptions, though. The regressions on entries related to my friends from my hometown and on music related entries yielded R^2^ values equal to 0.34 and 0.56, respectively, but other values were sometimes even virtually equal to zero.

**Table 4:** Mean Absolute Dating Error, R^2^ and Significant Predictors of the Regression Analysis with Absolute Dating Error as Dependent Variable.


	TOTAL SET	WORK	LITERATURE	HEALTH/MEANING	MY CHILDREN	TRAVELLING	PUBLIC EVENT	FRIENDS LEUVEN	MY WIFE	DREAMS	HOUSE/NEIGHBORS	EXTENDED FAMILY	MOVIE/TV/THEATER	MUSIC	EX ROMANTIC PARTNER	WEATHER/NATURE	FRIENDS/HOMETOWN

Mean	545	461	581	764	269	420	421	544	452	1364	322	249	422	773	922	1153	561

St. Dev.	839	639	790	990	479	883	887	728	645	1329	729	532	508	857	1440	1023	736

R squared	0.09	0.19	0.17	0.17	0.05	0.05	0.31	0.14	0.09	0.06	0.08	0.16	0.32	0.62	0.08	0.27	0.45

Adjusted	0.08	0.18	0.15	0.14	0.02	0.02	0.27	0.09	0.03	0	0	0.08	0.25	0.56	0	0.14	0.34

Entry Length	•	•		○													

Memory Age	•	•	•	•			•					•	•	•		•	•

Rehearsal	•	•	○								○			○		○	

Salience	•	•					○	○				○					○

Emot.lnv.														•			

Pleas.	•																

Intimacy													•				

Self-relat.				○										○			


*Note*: Significant contributions at the .01 level and at the .05 level in the regression are indicated, respectively with the symbols • and ○.

Considerably fewer variables contribute significantly to the predictions of the dating errors than in the regressions on the gradual retention scores. The length of the entry yields significance in only 3 out of the 16 regression analyses. The age of the memory is the most effective prediction variable, with a significant contribution in 10 of the 16 regressions. Rehearsal and salience are the two rating scales with most information in predicting the absolute dating error, though the number of regressions for which they reach significance is much lower again than in the analyses of the gradual retention scores. The significance pattern of the predictors seems again rather unsystematic, suggesting once more that autobiographical memories relating to different contents are stored somewhat differently.

## General Discussion

### Studying Autobiographical Memory From My Personal Diary Notes

Diary studies in which the investigator is the only participant are not common. One can assume that investigators who study their own memory are more motivated and more interested in memory than randomly picked persons. Immediately the question comes up whether the results of such studies can be generalized to ‘everybody’s memory’. However, participants in studies of autobiographical memory are typically college students, and, even more specific, psychology students in the USA, Europe or Japan. Besides the fact that the representativeness of this group of participants has been questioned (Henrich, Heine, & Norenzayan, 2010), materials used in group studies carried out in psychology laboratories seldom run over long periods of objectively verifiable events and over long time intervals before the testing of recall (White, 2002).

One might argue, as Wagenaar (1986) does, that the act of recording an event in a diary study makes the event stand out against the background of other events. However, the fact that keeping a diary has been a habit in my daily life for the past 4 decades, as well as the fact that I wrote the used material without any intention to use the entries in a memory study, make this criticism rather unfounded, I believe. (Besides, it is also possible that writing about an event in a diary results in more rapid forgetting, because the event is encoded anyway, be it in an external medium. See the photo-taking-impairment effect;Henkel, 2014.) The drawback of using my diary notes, sometimes written down concisely and in a hurry, sometimes more elaborately and detailed, is that the material had to be taken as it is, with no possibility for further control or adaptation. However, I believe that the advantages of the elaborate, verifiable test material, covering a very long period of time, outweigh these disadvantages and make the current memory study at least valuable and complementary to the more common group studies of autobiographical memory.^7^

### Measures of the Quality of My Memory

The dichotomous retention scores indicate that I remembered slightly less than two thirds of the events described in my diary notes. However, while questioning my memory for the described events, it became immediately obvious that it is often not at all clear whether I really remembered something. Memories can be vague, lacking even fundamental aspects of the events they are built on, but still leaving the feeling that these memories have not completely disappeared. Therefore, I felt more comfortable with the gradual retention scores, where I could indicate the extent to which I remembered the events.^8^ Even for the entries that I marked as remembered in the forced dichotomous score, the average 7-point gradual retention score (4.50) was hardly higher than the midpoint of the scale (which equals 4). One could interpret this finding as a response strategy showing that I was careful, avoiding extreme values if I did not remember all details of the event. Alternatively, it could mean that I recall hardly more than ‘half of the event’ (whatever that means). Anyway, this relatively low average score is evidence for gradually decaying memory traces, an interpretation that is further supported by the finding that there was a significant negative correlation between the gradual retention scores and the age of entries. Furthermore, this negative correlation remained when only looking at the events that I dichotomously judged as ‘remembered’ (r = -0.24, p < .001). The fact that dating error correlated higher (but negatively) with the gradual retention scores than with the all-or-none retention variable, as well as the finding that the gradual scores correlated higher than the all-or none variable with the six rating scales yields further support for a view on memory as a gradual, imperfect process.

In line with findings from the Catal and Fitzgerald (2004), Wagenaar (1986) and White (2002) diary studies, and opposite to the study of Linton (1978), the retention curve of my data showed a curvilinear, not a linear shape. This finding illustrates the rapid nature of the forgetting process. The nonlinear shape of the retention curve is also in line with laboratory studies that focused on many different materials and retrieval tasks, varying from nonsense syllables (e.g., Ebbinghaus, 1885) to cued recall (e.g., Averell & Heathcote, 2011) (See also Murre & Chessa, 2011, but see Radvansky, Doolen, Pettijohn, & Ritchey, 2022, for an alternative view.)

The retention curve that resulted from my study did not show a so-called reminiscence bump (Fitzgerald, 1988; Rubin, Rahhal, & Poon, 1998). The term is used to refer to a period when memory is remarkably better than before and after this period and such bumps have been observed when people are asked to name favorite films, music, books, important public events and so on. One might argue that this is not surprising, since I was 37 at the time of the first entries in the study, while the reminiscence bump typically covers the period when a rememberer was between the ages of 10 and 30. However, Rubin et al. explained the bump by pointing at the novelty of experiences and the effect such novelty has on memory. Moreover, Conway and Pleydell-Pearce (2000) mention the possibility of an ‘idiosyncratic retention bump’ for people who experience radical changes after the age of 35. The period covered by the entries in my study included quite some important first life experiences, like getting tenure, becoming a father and buying a house for the first time. The absence of a reminiscence bump suggests that such first time experiences were not sufficient to result in a temporally improved long term memory.

### What My Study Reveals About My Memory for Dating Events

While it is clear that we know the exact dates for *some* important and distinguished events, like marriage, birth dates or promotions, arguably no memory theory still assumes that *in general* the exact dates on which events occur are all stored directly in memory (Tzeng, 1976). In line with this, I was able to dig up the exact date for only a very small number of events (slightly over 2%). The small number of correctly dated events pleads against distance-based theories of memory for times and also against theories based on relative times of occurrence (Friedman, 1993). Instead, it seems that we use anchor points in estimating the date of events, that is, dating seems a reconstructive process of memory of time (e. g., Brown, Rips, & Shevell, 1985; Loftus & Marburger, 1983), combined with at least some order information.

In designing the study, I included a question on how I came to my best guess about the date the event took place. I thought it would be possible to write down whether dating was primarily based either a lifetime period, a general event, event-specific knowledge or just on a wild guess. (For the distinction between the first three, see Conway & Pleydell-Pearce, 2000.) However, I gave up this plan after a few days because I found it too difficult. Trying to categorize my associations after reading an entry in one of these structuring sorts of periods seemed too random and unreliable.

Reflecting further on the dating process after the study was finished, I remember several different strategies. As mentioned above, for some events an exact date was immediately activated from memory. For instance: I ‘know’ the date of the events that took place the day my father died (and the day after that). Dating other events required more abstract knowledge, combined with inferential reasoning. An example is meeting Jimmy Carter in New York. I remember the event vividly, but dating this event required me to think how many summer holidays I remember taking place after that particular family trip to the USA. Still other events could only roughly be dated after thoughts like, for instance, “it must have been not long before we moved to the country side”. It is then not clear though, whether dating is based on a specific event (i.e., moving our belongings from Leuven to the countryside) or on a lifetime period (i.e., the time we spent in Leuven as opposed living in the countryside). Yet another set of events, remembered or not-remembered, gave me no clue at all as to when it happened. In those cases, my best estimate was not more than a wild guess. Summarizing, though I sometimes was able to spell out retrieved information that somehow guided the dating process, it seemed very hard to find structure in these thought processes. Exposing the principles behind these processes require additional research. However, the results show that I do have metacognition on the dating process, as there was a significant correlation between my confidence in and the accuracy of my date estimates.

On average, the difference between the correct and the estimated date was a bit over one and a half year. As in Wagenaar (1986) and White (1982), the number of events for which I overestimated the age was about equal to the number of underestimations. As Wagenaar remarked, this symmetry contrasts with the forward telescoping phenomenon (Loftus & Marburger, 1983), which states that the time elapsed since salient events is underestimated. To further investigate whether telescoping was present in my memory when controlling for salience, I calculated the dating error (negative if estimated too recently; positive if estimated too far in the past) for the different values of rated salience and found no convincing evidence for telescoping: the average dating errors for ‘once in a life time’, ‘once every 15 years’, ‘once every 5 years’, ‘once in a year’, ‘once in a month’ and ‘once a week’ were –440, 197, 0, 80, –78, and –404 days, respectively.

Over or underestimation did not correlate with pleasantness, nor with emotional involvement. The modest correlation between the age of the event and the dating error, r = 0.17, was comparable to similar correlations reported in the diary study of Catal and Fitzgerald (2004) which spanned a comparable time period of 20 years: r = .06 and r = .16 for the female and the male participant, respectively.

### Predictive Value of the Ratings

Five of the six rating scales correlated significantly with the gradual retention scores. Only intimacy did not, despite the reasonable test-retest reliability of these ratings. Even with a relatively low estimated reliability, the self-relatedness ratings did yield a significant correlation with gradual retention, but only because of the very large number of pairs in the calculation (N = 2542). This finding is in line with the lack of correlation between self-relatedness ratings and retention reported in White’s (1982, 1989, 2002) study.^9^

Similar to Wagenaar’s (1986) autobiographical memory experiment, I found significant, but moderate correlations between gradual retention and the ratings of salience, with more salient events (i.e., events that happen only once every 5 or 15 years, or once in a lifetime) being remembered better. After controlling for the relatively low reliability of the salience ratings (estimated to be 0.48), the correlation of salience with the gradual retention scores and with the dating errors becomes 0.25 and –0.14, respectively, which is still not very high.

Contrary to what Talarico, LaBar and Rubin (2004) reported from a group study with college students, in my data the relation of gradual retention with salience was clearly lower than that with pleasantness. My gradual retention scores thus showed a tendency to increase as my pleasantness scores increased.

Again in line with Wagenaar (1986), I found a modest, but significant correlation between gradual retention and emotional involvement. However, as Linton (1982) argued, ratings of emotionality at the time of retrieval may differ from experienced emotionality when the event actually happens.

The rating scale that correlated, by far, highest with gradual retention was event rehearsal: the more I remember talking (and ruminating) about an event, as judged on the rating scale, the higher the gradual retention score. Similar findings were reported in Walker, Vogl, and Thompson (1997) and by Linton (1978). The event rehearsal ratings correlated even higher with gradual retention than the amount of information presented, as measured by the length of the diary entry.

The pattern of correlations of the six ratings with dating error was more or less the same as that with the gradual retention scores, the difference being that all correlations were considerably lower and thus fewer correlations yielded significance.

When all ratings, together with entry length and the age of the event, were combined as predictors in regressions, the gradual retention scores again turned out to be much better predictable than dating. Almost 40% of the variance in de gradual retention scores could be predicted, compared to only 9% of the absolute dating errors. In both analyses, most ratings contributed significantly, with the exception of intimacy and emotional involvement. Although emotional involvement also correlated substantially with gradual retention and with dating errors, the effect of this variable as predictor disappeared when combined with event rehearsal in the regression analyses. The absence of a significant contribution of emotional involvement may come as a surprise at first sight, but it can easily be interpreted in light of the finding that most people share their emotional experience. Social sharing of emotions has been shown to be common practice (Rimé, 2009; Rimé, Mesquita, Philippot, & Boca, 1991; Rimé, Philippot, Boca, & Mesquita, 1992) and my results suggest that the extent of social sharing and rumination – two processes that determined my ratings of event rehearsal – flood the influence of emotional involvement on retention.

The finding that event-rehearsal was the best predictor of my memory leads to the question why I more often discussed and thought about certain events. This important question, however, cannot be answered with the available data from this study.

### Separate Content Category Analyses

When Wagenaar (1986) compared the predictive value of the ‘when’, ‘where’, ‘who’, and ‘what’ cues, he found that the content of a memory (i.e., the ‘what’) was by far the most effective cue. A similar finding was reported by Catal and Fitzgerald (2004). Surprisingly, however, none of the single participant diary studies so far distinguished between different contents of personal memories when analyzing the data. Since I sorted all entries into 16 content categories that have been important to me in making diary notes over the past four decades, I was able to perform separate analyses for these categories. (I have no reason to assume that the same 16 categories are equally meaningful to other people. However, my plea for further studying different kinds of memory content in autobiographical memory does not depend on the specific choice of the categories.)

There was a large variability in the average gradual retention scores of the 16 categories, as well as in the corresponding average dating errors. Dreams, for instance, can be very emotional when waking up and can leave a pervasive impressive during the following day, which sometimes caused me to entrust it to my notes, but it turns out that I forgot the dreams quite easily. Likewise, weather conditions, like an unexpected snow shower that prevented me from attending an important meeting, can have disruptive momentary consequences, but don’t seem to leave long-lasting memories. Also, a dispute with my wife can cast a shadow on an evening, but will mostly have disappeared in oblivion a few weeks later. However, the last conversation I had with my deceased mother, or travel experiences, like a first time visit to an exotic country, leave remaining traces. That such content categories are systematically differently stored is also clearly shown in that the average gradual retention scores of the 16 categories correlate highly with the corresponding mean dating error (r = –.74, p < .01).

How can the differences in memory strengths within the different content categories be explained? Again, the most important predictor was event rehearsal. The extent to which I talked about the events afterwards contributes significantly in predicting the gradual retention scores in 15 of the 16 categories. Unsurprisingly, the time past since the event took place also exerts a considerable influence in all content categories. But besides these two dominant predictors, other predictors did or did not contribute in the prediction of memory strength depending on the content category. Pleasantness, for instance, determines the gradual retention scores significantly for entries related to my work, public events, extended family, movies/TV/theater and ex romantic partners, but not for entries of the remaining content categories. And intimacy and emotional involvement contribute (over and above the influence of the other predictors) in explaining the differences in memory strength related to my children. Since the error in dating the events within categories is much more difficult to explain (as was obvious from the much lower R^2^ values of the corresponding regressions), few predictor variables yield significance. In general, though, the pattern is similar, but much more mitigated.

Summarizing, the results of my study are rather similar to older attempts to study autobiographical memory in single participant diary studies. Such studies have been criticized because there is no guarantee that the memory of the researcher functions in the same way as that of others. However, the similarities between the results of Catal and Fitzgerald’s (2004), Linton’s (1975, 1978), Wagenaar’s (1986), and White’s (1982, 1989, 2002, 2020) studies cast doubt on the validity of this criticism. I hope that scholars of autobiographical memory will continue to investigate time and energy in diary studies, which can cover equally long time periods, with similarly ecologically valid material, but especially the variation in retention for the different content categories, neglected in earlier studies, deserves further investigation.

## Data Accessibility Statements

The author and the single participant in this study agree that the raw material (i.e., the text of the diary notes) are too personal to be open for readers to inspect. Furthermore, to avoid law suits from people who figure in the diary, especially colleagues, it seems best to restrict openness to the excel file on the page of the Open Science Foundation https://osf.io/ez6rj/.

## Footnotes

Because of the sometimes very personal character of the content, I did not want anyone else to read the diary notes.

I thank Sven Peremans for programming the application.

For all events in his study, recorded by the cue words what, where, when and who, Wagenaar (1986) added a ‘critical detail’ that was selected such that successful retrieval of the event from memory given *who, what, where*, and *when* would almost guarantee the recall of the detail.

Note that the idea for the study occurred to me late December 2020, meaning that the last included entries were written down while I was aware of the planned study.

I did not distinguish between making a mistake and simply not having the opportunity to infer a capitalized place or person name because no capitalized proper name occurred in the entry. In hindsight, this is a missed opportunity.

Restriction of range may have affected the correlations between the different variables to some extent. The averages of the ratings over the 2543 entries for salience, emotional involvement, pleasantness, intimacy, event rehearsal and self-relatedness were, respectively, 3.05, 2.29, 2.59, 1.20, 2.05, and 1.81. The corresponding standard deviations were, respectively, 0.84, 1.27, 1.57, 0.64, 1.24, and 1.14. Note also that correlations with the ratings are depending on their reliability and that especially the reliability of self relatedness and of salience were quite low, while those of pleasantness and event rehearsal were relatively high.

Note that the study of Catal and Fitzgerald (2004), husband and wife, is not a single participant study, as both spouses tested their memory for events written down over a period of two decades by only one of them. This study thus does not suffer from some of the criticism described above.

The gradual retention scores I used are somewhat similar to the Know-Remember scale, used by Hyman, Gilstrap, Decker and Wilkinson (1998) and by Catal and Fitzgerald (2004), but there is an important difference. In the way I used the scale, the minimum value of 1 refers to events that were completely forgotten, while a minimum value in the Know-Remember scale indicates that one recognizes the event to have occurred but without any recollection or clear memory of the event. Therefore, my score values are not comparable to those of the Know-Remember scale.

As a reviewer remarked, if predominantly events that were highly related to self were recorded in my diary, then the modest correlation between the self-relatedness ratings and retention is not surprising.
